# Comparison of Factors Contributing to Developmental Attainment of Children between 9 and 18 Months

**DOI:** 10.2188/jea.JE20090177

**Published:** 2010-03-05

**Authors:** Shunyue Cheng, Tadahiko Maeda, Zentaro Yamagata, Kiyotaka Tomiwa, Noriko Yamakawa, Japan Children’s Study Group

**Affiliations:** 1 Research Institute of Science and Technology for Society, Japan Science and Technology Agency, Tokyo, Japan; 2 The Institute of Statistical Mathematics, Research Organization of Information and Systems, Tokyo, Japan; 3 Department of Health Sciences, School of Medicine, University of Yamanashi, Chuo, Yamanashi, Japan; 4 Graduate School of Medicine, Kyoto University, Kyoto, Japan; 5 Clinical Research Institute, Mie-chuo Medical Center, National Hospital Organization, Tsu, Japan

**Keywords:** child development, environmental factors, parenting stress, prospective study

## Abstract

**Background:**

Little is known about how contributing factors of development change during early childhood in Japan. The aim of this study was to investigate the factors that contributed to the developmental attainment of children between 9 and 18 months of age using prospective longitudinal data from a developmental cohort study.

**Methods:**

We used data from observations at 3 time points (at infant age of 4, 9 and 18 months) in the Japan Children’s Study. Mothers were administered questionnaires that requested information about their child’s perinatal outcomes, temperament, family structure, family income, parental education, parenting stress, and child-rearing environment at home. At 9 and 18 months, mothers completed the Kinder Infant Development Scale to evaluate their child’s development.

**Results:**

A total of 284 children were available for analysis. Female children and children having siblings had higher probability of attaining developmental norms at 18 months than male and only children. Birth weight, gestational age, and temperament were associated with development at 9 months, but the effects of gestational age and temperament on development disappeared at 18 months. Stimulation from the mother at 9 months was not only related to development at that age but also promoted development at 18 months.

**Conclusions:**

Our findings suggest that the role of family environmental factors such as early mother’s stimulation and sibling’s existence in development during early childhood might become more important as the child gets older.

## INTRODUCTION

Many risk factors have been implicated in the etiology of developmental impairment in young children, including biological, social, and environmental influences.^1^^,^^2^ Although biological risks are important determinants of all domains of development, psychosocial risks can also adversely affect cognitive and social–emotional competence. The quality of early childhood care has a clear biological impact on the developing brain and has long-term implications on the child’s development and psychological health.^3^ The Committee on Integrating the Science of Child Development^4^ asserted that the first few years of life are particularly important because vital development occurs in all domains. The relative weights of risk factors change during the early years of life, biological factors becoming less important and psychosocial ones gaining influence.^5^^,^^6^

Previous studies have highlighted the importance of parenting activities, parental psychological stability, and socio-economic factors in a child’s developmental attainment.^7^^,^^8^ The family provides the significant care and stimulation required for the child’s growth and development, especially during early childhood. Paradoxically, in the family environment, children either receive protection or are exposed to risks to their development. Reported risk factors are often associated with low socio-economic status and weak family ties,^9^ which may impair a child’s ability of problem-solving as well as language, memory, and social skills. Many authors agree that maternal schooling has an impact on children’s development through factors such as environment structuring, parents’ expectations and practices, experiences with cognitive stimulation materials, and variety in daily stimulation.^9^^,^^10^

Currently, in Japan, children with biological risk factors are supposed to be screened by primary medical care providers and receive early referral to developmental intervention programs. However, many children with poor development that lack obvious risk markers may not receive the benefit of early identification and intervention. Furthermore, there are many studies concerning the physical health but relatively few concerning the mental health of children in Japan. Examination of the factors that support development during infancy is important to the design of effective interventions for infants at a risk of poor development.

The present study was undertaken to identify which aspects of family factors—especially learning stimulation, parenting stress, and family function—contribute to the children’s development and how role of these factors change from 9 to 18 months of children’s age based on a longitudinal cohort project, the Japan Children’s Study (JCS).

## METHODS

### Participants

Data from the JCS project, recorded at infant ages of 4, 9, and 18 months were used in this study. The JCS project is a prospective developmental cohort study started in 2005 by the JCS research group at three study sites in Japan: Osaka, Mie, and Tottori. The purpose of the project is to describe the development of sociability in children and investigate factors promoting this development. Infants who were born between August 2004 and April 2006 and their parents were recruited from the above three sites. The recruitment of subjects is described in detail elsewhere.^11^ Parents completed self-administered questionnaires sent to them before the observations that were made at 4, 9, and 18 months of the child’s age and brought them to the laboratory. A total of 479 families participated in the baseline assessment and children whose mothers completed the questionnaires at baseline and continued to participate in the observation at 9 and 18 months (403) were eligible for this study. Since the questionnaires were revised at the baseline assessment, there were complicated missing data on all relevant items. Because this was not the results of response willingness on the part of the participants, it is assumed that the missing data were missing at random and therefore not biasing our findings. A total of 284 mother–child dyads with completed questionnaires on all relevant items were available for statistical analyses.

### Measures

Child development outcomes were assessed using the Kinder Infant Development Scale (KIDS)^12^ when the children were 9 and 18 months of age. KIDS is a developmental screening scale that is convenient to use and can be easily completed by the parents. It was standardized in 1989 using 6000 Japanese children aged 0–6 years, and the validity of the scale was confirmed.^12^ The scale is composed of subscales for the developmental domains of physical motor, manipulation, language, social, and feeding. The total score is used as a comprehensive evaluation of the infant’s development. In this study, KIDS type A (for infants aged 1–11 months) and type B (for children aged 12–35 months) were used to assess the children’s development at 9 and 18 months, respectively. Each item in both types of KIDS has the options “pass” and “fail.” These two responses were scored 1 and 0, respectively, and the scores for all items in each subscale were summed to obtain the full developmental score; a higher score reflected a higher level of development. Values of Cronbach’s alpha were 0.86 and 9.1 for the comprehensive development scores at 9 and 18 months, respectively, in this sample.

Mothers completed the Evaluation of Environmental Stimulation—Short version (EESS)^13^^,^^14^ scales to assess the child-rearing environment when their children were aged 9 and 18 months. The scale items were chosen based on Home Observation for Measurement of Environment (HOME)^15^ and contained 13 items clustered into four subscales: human stimulation (five items), avoidance of restriction (two items), social stimulation (three items), and support (three items). For the purpose of this study, two subscales concerning learning stimulation were used: human stimulation, which is mainly provided from the infant’s mother (playing with the child, reading books or stories to the child, singing songs to or with the child), and social stimulation, which is given through activities related to the environment (take child shopping, to the park, or to a relation’s or friend’s house). Responses ranged from 1 (“rarely”) to 4 (“almost every day”), and the scores were summed for both subscales, a higher score reflecting a higher level of stimulation. Cronbach’s alpha was 0.50 for human stimulation and 0.53 for social stimulation in this sample. These values of alpha indicate that internal consistency fell short of a satisfactory level, but they reflect a shortage in the number of items rather than any inadequacy of the items themselves, and we decided to use these scores as they were.

When the infants were 9 months of age, the mothers completed the Japanese version of the Family Apgar scale^16^ to assess their family functioning. This five-item scale was designed to assess perceived family support in the domains of adaptation, partnership, growth, affection, and problem solution. The statements focus on the emotional, communicative, and social interactive relationships between the respondent and his or her family. Responses were scored from 0 (“strongly disagree”) to 2 (“strongly agree”), and higher scores indicated greater family function. Cronbach’s alpha for this sample was 0.85.

A rearing-related stress (RRS) questionnaire^17^ was included to assess parenting stress and was administered to the mother when the child was 9 months of age. The questionnaire was originally developed to assess the degree of stress experienced by parents in two domains: child RRS and maternal RRS. The latter consists of 10 items that contribute to maternal stress and was used in this study. Examples are: “I don’t know how to treat or rear a baby,” “My husband does not pay attention to our baby,” and “I feel confused because there is too much information about child rearing.” There are four response categories, from 1 (“Strongly disagree”) to 4 (“Strongly agree”), higher scores indicating higher parenting stress. Cronbach’s alpha for this sample was 0.77. We defined scores at or above the 75th percentile as high-level parenting stress and below the 75th percentile as low-level parenting stress.

When the infants were 9 months of age, the mothers completed a 42-item questionnaire on child temperament developed for this study.^18^ Selection of these 42 items was based on previous studies.^19^^,^^20^ The questionnaire measured seven dimensions of temperament: taste reactivity, audiovisual reactivity, negative emotionality, frustration tolerance, persistence in task situation, approach to new situations or people, and behavioral rhythmicity. Each dimension consisted of six items. Responses were scored as Likert scale ratings ranging from 1 (“almost never”) to 6 (“almost always”), describing the child’s standard day-to-day behavior. Internal consistency of these scales was reported by Ogura.^21^ We used two dimensions in this analysis where Cronbach’s alpha was above 0.60: persistence in a task situation (0.61) and the approach to new situations or people (0.85). Higher scores indicate greater persistence in the task situation and an easier approach to new situations.

Information on parents’ ages and levels of education, annual family income, type of family, and the children’s sex, birth weight, number of children, and gestational age (number of full weeks) was obtained from a questionnaire completed by the mother when the child was 4 months of age.

### Statistical analysis

For each categorical variable we calculated frequencies and percentages and for each continuous variable we calculated the mean and standard deviation at 9 and 18 months to summarize participant characteristics.

Logistic regression was used to explore the factors that contributed to developmental attainment at 9 and 18 months of age. The KIDS comprehensive developmental scores were recorded as binary variables, a value of 1 representing “pass” (attaining the norm) and 0 representing “fail” (not attaining the norm). According to the KIDS manual, the pass rate is approximately 65% in the population. To compare factors related to developmental attainment between 9 and 18 months of age, two logistic regression analysis models were calculated. In the first model, we entered developmental status at 9 months as the outcome variable and the factors at baseline and 9 months as the independent variables. In the second model, developmental status at 18 months was the outcome variable; the current stimulation variables were added to the same variables in the first model and entered simultaneously as independent variables.

The child’s sex, birth weight (low or normal), gestational age (preterm or full term), postnatal age (days), two dimensions of temperament (persistence and approach), mother’s education level (greater than high school or high school or lower), type of family (nuclear or extended), household income (>4 million or ≤4 million Japanese yen per year), number of children (one, or two or more), Family Apgar score (<6 or ≥6), mother’s parenting stress (high or low), and mother’s environmental stimulation were included in the models as independent variables. Odds ratios and 95% confidence intervals were calculated. All analyses in this study were performed using SPSS version 17.

### Ethics

The study protocol was approved by the Ethics Review Committees of the collaborating research institutes (Osaka City General Hospital, Mie-chuo Medical Center, and Faculty of Regional Sciences, Tottori University) and the Ethics Review Committee of the Research Institute of Science and Technology for Society, Japan Science and Technology Agency, which based their approval on the “Guidelines Concerning Epidemiological Research” promulgated by the Japan Ministry of Education, Culture, Sports, Science and Technology and Ministry of Health, Labour and Welfare.

## RESULTS

Approximately 89% were nuclear families, 75% of the mothers had completed post-high school education and 70% of families had an annual income of ≥4 million Japanese yen. The percentage of children with low birth weight (<2500 g) was 9.2%, and 4.9% of the children were delivered preterm (<37 weeks). Mean postnatal age was 9.4 months (282 days) and 18.5 months (556 days) for the time points of observation, respectively (Table 1).

**Table 1.** Summary of participants characteristics and description of variables included analysis (*n* = 284)

Variables	*n* (%)	Mean (SD)	Range
Child factors			
Sex (female)	142 (50)	—	—
Birthweight (<2500 g)	26 (9.2)	2988.7 (390)	1278–4156
Gestational age (<37 weeks)	14 (4.9)	39.0 (1.4)	31–42
Postnatal age at 9 mo (days)	—	282 (7.6)	265–333
Postnatal age at 18 mo (days)	—	556 (7.3)	535–588
Temperament: Persistency	—	22.0 (5.9)	6–36
Approach	—	25.6 (6.8)	6–36
Family factors			
Number of child (only)	157 (55.3)	1.6 (0.7)	1–4
Family type (nuclear)	254 (89.4)	—	—
Maternal education (≤high school)	70 (24.6)	—	—
Family income (≤4 million per year)	87 (30.6)	—	—
Family APGAR at 9 mo (<6)	77 (27.1)	7.9 (2.2)	0–10
Parenting stress at 9 mo (high)^a^	91 (32.0)	19.5 (4.2)	10–34
Mother’s stimulation at 9 mo	—	5.0 (1.9)	2–8
Environmental stimulation at 9 mo	—	5.0 (1.5)	2–8
Mother’s stimulation at 18 mo	—	13.0 (2.2)	5–15
Environmental stimulation at 18 mo	—	10.0 (2.4)	3–15

^a^Scores over 75th percentile as a high level of stress.

For comprehensive development, about 66% and 83% of children attained the norm at 9 and 18 months, respectively, according to the cut-off points stated in the KIDS manual. For the outcome at 9 months, children with low birth weight (OR = 0.36, 95% CI: 0.13–0.97) and preterm delivery (OR = 0.18, 95% CI: 0.05–0.72) had a significantly lower probability of attaining the developmental norm compared with children with normal birth weight and full-term delivery, even when adjusted for other factors (Table 2). Children with higher scores for persistency (OR = 1.11, 95% CI: 1.06–1.16) and maternal stimulation (OR = 1.74, 95% CI: 1.17–2.59) had a higher probability of attaining the developmental norm. For the outcome at 18 months, compared with male children, female children had a 2.3 times higher odds of attaining the developmental norm. Low birth weight (OR = 0.25, 95% CI: 0.09–0.72) had a negative impact on developmental attainment. Compared with single children, children who had siblings had a higher chance of attaining the developmental norm (OR = 2.09, 95% CI: 1.01–4.32). Children who received more frequent stimulation from their mothers had a higher chance of attaining the developmental norm even after adjustment for current stimulation variables. When mothers provided a more stimulating environment to their children, their children tended to have a higher chance of attaining the developmental norm at 18 months (OR = 1.58, 95% CI: 0.95–2.65), but the difference was not statistically significant at the 5% level (*P* = 0.07). The influence of the child’s gestational age and temperament on development disappeared at 18 months. The other family environmental factors, comprising family type, maternal education, family income, family function, and maternal parenting stress, were not related to development at either time point.

**Table 2.** Multiple logistic regression analysis for related factors on development attainment at 9 and 18 months

Variables	9 months OR (95% CI)		18 months OR (95% CI)	
	
	Unadjusted	Adjusted^b^	Unadjusted	Adjusted^c^
Child factors				
Female	1.25 (0.76–2.04)	1.37 (0.78–2.39)	2.05 (1.08–3.91)	2.28 (1.11–4.68)
Low birthweight	0.41 (0.18–0.92)	0.36 (0.13–0.97)	0.34 (0.14–0.81)	0.25 (0.09–0.72)
Preterm infants	0.19 (0.06–0.62)	0.18 (0.05–0.72)	0.49 (0.15–1.62)	0.52 (0.11–2.39)
Postnatal age	1.03 (0.99–1.06)	1.04 (1.00–1.08)	0.97 (0.94–1.01)	0.99 (0.94–1.03)
Temperament: Persistency	1.09 (1.04–1.14)	1.11 (1.06–1.16)	1.05 (0.99–1.10)	1.05 (0.99–1.11)
Approach	0.96 (0.93–1.01)	0.96 (0.92–1.01)	0.97 (0.93–1.02)	0.97 (0.93–1.02)
Family factors				
Have siblings	1.04 (0.64–1.70)	1.08 (0.61–1.88)	1.43 (0.76–2.71)	2.09 (1.01–4.32)
Nuclear family	0.35 (0.13–0.95)	0.35 (0.11–1.10)	0.74 (0.25–2.22)	0.79 (0.23–2.67)
Maternal education (≤high school)	0.84 (0.48–1.47)	0.84 (0.43–1.63)	0.97 (0.47–1.99)	0.94 (0.41–2.15)
Family income (≤4 million per year)	0.85 (0.50–1.43)	0.75 (0.41–1.38)	1.39 (0.68–2.82)	1.36 (0.61–3.00)
Family APG at 9 mo (<6)	1.02 (0.59–1.78)	1.09 (0.58–2.06)	1.13 (0.56–2.31)	1.28 (0.57–2.87)
Parenting stress at 9 mo (high)^a^	1.14 (0.69–1.87)	1.27 (0.71–2.26)	0.91 (0.48–1.72)	0.81 (0.39–1.66)
Mother’s stimulation at 9 mo	1.59 (1.12–2.26)	1.74 (1.17–2.59)	1.69 (1.10–2.62)	1.97 (1.17–3.30)
Environmental stimulation at 9 mo	1.15 (0.73–1.81)	0.90 (0.53–1.52)	1.32 (0.76–2.30)	1.15 (0.61–2.16)
Mother’s stimulation at 18 mo			0.98 (0.60–1.58)	0.69 (0.43–1.10)
Environmental stimulation at 18 mo			1.66 (1.05–2.61)	1.58 (0.95–2.65)

^a^Scores equal to or more than 75th percentile as a high level of stress.

^b^Adjusted for all variables excluded two variables: the mother’s and environmental stimulation at 18 months.

^c^Adjusted for all variables in the model.

## DISCUSSION

This prospective longitudinal study examined factors contributing to development at 9 and 18 months of age and also investigated how these factors changed between these time points. The results demonstrated that in addition to the child’s biological factors, the child’s persistency and cognitive stimulation from the mother were significantly associated with developmental attainment at 9 months. Female children, children having siblings, and children receiving frequent stimulation from their mother had higher probability of attaining the developmental norm at 18 months. These findings reinforce the results of previous studies showing that the importance of risk factors changes over time^6^^,^^22^ and supports the notion that environmental factors play an increasing role in the development of children as they grow older.

The results of our study highlight the importance of both human and environmental stimulation for the development of young children. Experiencing more stimulation from the mother in infancy increases the chance of attaining concurrent and later developmental norms. This result is consistent with that of previous studies on the association between environmental stimulation and cognition, and demonstrates that mothers who provide more stimulation to their infants through a variety of perceptive experiences with people, objects, and symbols contribute to their children’s cognitive development and to favorable outcomes.^23^ Moreover, in our data, this influence became stronger at 18 months, even when adjusted for concurrent maternal stimulation. It is important to note that early maternal stimulation is likely to have a stronger influence on a child’s development than concurrent stimulation. The initial years of an infant’s life represent a critical period of brain development^24^ and are therefore an ideal time for interventions intended to prevent the later development of psychopathology and behavioral and developmental abnormalities.^25^ Our results are in line with previous studies and indicate the important influence of early quality of parenting practices on subsequent development. Contrary to the results regarding the mother’s stimulation, concurrent environmental stimulation tended to have stronger effects than maternal stimulation on developmental attainment at 18 months. This implies that providing children with a variety of enriching out-of-home experiences during their second year of life is probably more effective in stimulating their development, since they acquire more skills when interacting with their surroundings.

There is evidence that a male and a female child’s physical environments differ in a gender-related manner and that the two genders are often exposed to different linguistic environments according to the gender typing of their play activities.^26^^–^^28^ However, some studies reported that a gender difference in communicative development is not obvious as early as in the second year of life as children do not develop speaking ability at this age.^29^^,^^30^ Our findings are consistent with previous results showing that female children are faster in reaching developmental milestones than male children, though we observed a sex difference at 18 but not 9 months. Having siblings significantly increased the chance of attaining the developmental norm compared with being a single child. A study conducted by Arone^31^ suggested that having siblings may be a promoting factor for mental health, possibly by offering a child an opportunity to develop his or her social problem-solving skills and ability to get along with other children. Our results indicate that as children grow older and their communication ability increases, the sibling’s role also becomes more important.

The impact of the child’s persistency and gestational age on development at 9 months weakened or disappeared by the age of 18 months. Maternal education and family income were not associated with development in our data. Numerous studies have documented that poverty and low parental education are associated with lower levels of cognitive development.^9^^,^^32^^–^^34^ Our results might be explained by the fact that our study sample was relatively homogeneous in both maternal education and family income compared with previous studies. Maternal parenting stress and family function were also unrelated to developmental attainment at any time point in our study. Further follow-up observations from this prospective study are needed if we are to understand the associations between maternal parenting stress, family function, and child development attainment.

The main limitation of this study is that all measures relied on the mothers’ reporting, and therefore, the validity of the results is limited by the accuracy of the parents’ responses. Another limitation is that since the child development outcomes were assessed with a domestic Japanese instrument, an international comparison of our results is not possible. In addition, for the outcome at 9 months, the children’s temperament, parenting stress, family function, and learning stimulation variables were measured at the same time as the outcomes. Simultaneous evaluation of these variables with the outcomes makes it impossible to evaluate causal relationships, allowing only the verification of association between these variables. Additional longitudinal data analyses are required to further investigate the causal relations of these exposures. Nevertheless, we believe that these findings indicate a promising direction for future research on maternal parenting behaviors and children’s development.

Despite this limitation, this is the first longitudinal cohort study of mental health in early childhood in Japan, and our findings might provide important suggestions for intervention studies in future to promote child development at different ages. The JCS sample, although not completely representative of the Japanese population, is useful in that the cohort was selected from different areas. Additionally, our analysis controlled for a wide range of potential confounders, including the child’s birth weight, gestational age, temperament, maternal education, socio-economic status, and other familial factors.

The overall results of this study are consistent with those of previous studies, suggesting that social and environmental factors tend to become stronger determinants of children’s development as they get older (5,6). Our findings clarified that the impact of family factors, including maternal cognitive stimulation and the existence of older siblings in early childhood, becomes stronger with the increasing age of the child. The results of our study suggest the need for preventive or psychoeducational interventions for expectant parents or young families, focused on helping them in their parenting practice of providing cognitive stimulation as their children grow and change.

More information is needed to clarify the long-term relationship between these factors and development. These are the issues we will focus on in subsequent stages of our longitudinal study.
